# Vicarious Trauma Among a Sample of European Nurses: Using Qualitative Findings From a Mixed Methods Study to Develop an Organisational Leadership Guideline

**DOI:** 10.1155/jonm/9888206

**Published:** 2026-08-22

**Authors:** Kristine Nicki Annunziata, Elizabeth Curtis, Catherine Comiskey

**Affiliations:** ^1^ School of Nursing and Midwifery, Trinity College Dublin, Dublin, Leinster, Ireland, tcd.ie

**Keywords:** addiction nursing, evidence-based guidelines, organisational leadership, vicarious trauma

## Abstract

**Aim:**

This paper presents the qualitative findings of a European mixed‐methods study exploring vicarious trauma (VT) among nurses working in addiction services. Its primary objective is to share with readers the newly developed organisational leadership guidelines in the hope of mitigating the impact of VT.

**Background:**

VT is a recognised occupational hazard for nurses working with traumatised populations, particularly in addiction services, where proactive and supportive leadership is critical in fostering resilience and safeguarding staff wellbeing. However, organisations have failed to implement proactive strategies to identify, manage or prevent VT. This study addresses this gap by examining nurses’ lived experiences of VT and the role of leadership in reducing the impact of VT. These insights together with results from the quantitative phase of the study, alongside a literature review, were used to develop the guidelines.

**Method:**

Fifteen participants from the study’s quantitative phase were interviewed using semistructured questions. Data were analysed thematically and an inductive coding approach ensured findings were grounded in participants’ narratives.

**Results:**

Five core themes emerged are as follows: (1) positive growth associated with VT, (2) recognising negative consequences, (3) the role of organisational leadership, (4) support systems and (5) the evolving identity of addiction nurses. Leadership was recognised as functioning either as a risk or as a protective factor, depending on its form and application.

**Conclusions:**

The qualitative findings of the study were used to develop the empirically informed organisational leadership guidelines to address VT in nursing. These guidelines represent a significant step forward in equipping healthcare organisations to protect their staff through proactive, structured organisational leadership interventions. Examples of these include awareness, education and support.

**Implications for Nursing Management:**

This study underscores the critical role of organisational leadership in either exacerbating or alleviating VT. By implementing evidence‐based guidelines that promote awareness, shared decision‐making, emotional support and trauma‐informed practices, nurse leaders can improve staff wellbeing, reduce stress and enhance service delivery in addiction service settings.

## 1. Introduction

Vicarious trauma (VT) among nurses working in addiction services remains underexplored despite its significant implications for both staff wellbeing and patient care. The concept of VT was first conceptualised by Pearlman and McCann in 1990, describing the psychological impact on individuals who are indirectly exposed to traumatic events through their professional roles. While VT has been acknowledged in various healthcare settings, its application to addiction services remains limited, despite nurses’ frequent exposure to complex trauma, relapse, and psychosocial adversity [1, 2].

Existing evidence suggests that addiction nurses are at moderate to high risk of experiencing negative emotional and psychological outcomes associated with repeated trauma exposure, including emotional exhaustion and compassion fatigue [2, 3]. However, research has largely focused on individual‐level coping strategies, with comparatively limited attention to organisational and leadership influences on VT. Organisational leadership within healthcare organisations is widely recognised as a critical factor in either exacerbating or alleviating the impact of VT [2]. Effective leadership and supervision are crucial in addressing and preventing VT among staff, given that poor leadership can significantly increase nurses’ vulnerability to its effects [2]. For instance, nurses who reported insufficient leadership support, unclear decision‐making communication or a lack of proactive organisational strategies faced a markedly higher risk of VT or burnout [2–5]. Key organisational factors, including a lack of clarity in the organisation’s mission, infrequent trauma‐focused meetings and inadequate leadership involvement in mitigating trauma, were also found to contribute significantly to the heightened risk of VT.

Despite this, there is limited evidence on how addiction nurses themselves perceive leadership support and what organisational strategies they consider most effective in reducing VT. Therefore, the aim of the qualitative phase of the study was to explore the lived experiences of nurses working in addiction services with regard to VT. To achieve this, the study sets out to understand nurses’ view about the proactive factors that can alleviate the impact of VT, as well as their views on leadership and its role in minimising the risks associated with VT. In addition, an outcome of the study was to provide the first evidence‐based empirically informed organisational leadership guidelines to support nurses from the negative effects of VT, thereby promoting a healthier work environment and improving overall patient care in addiction services.

## 2. Methods

### 2.1. Design

This study adopted an explanatory sequential mixed‐methods design, comprising initially a quantitative phase, followed by a qualitative phase. The quantitative phase focused on identifying the prevalence of VT among nurses working in addiction services, as well as the associated risk and protective factors. The qualitative phase aimed to provide deeper insights into the lived experiences of nurses, focussing on their encounters with VT and the role of leadership in mitigating its effects. The qualitative results were used to complement and further explore the findings from the quantitative phase, allowing for a more comprehensive understanding of the challenge. Although the study employed a mixed‐methods design, this paper focuses on the qualitative findings, which, together with the quantitative results, informed the development of the preliminary evidence‐informed organisational leadership guidelines.

### 2.2. Setting and Sample

The study was conducted across several addiction services in Europe. At the end of the quantitative phase, participants were invited to indicate if they wished to take part in the qualitative phase by leaving their email addresses. Of the 30 who volunteered, 15 were purposively selected to ensure coverage across different European regions and a balanced demographic. Selection prioritised maximum variation to capture a broad range of perspectives rather than statistical representativeness. The sample size of 15 participants was considered appropriate for this qualitative study within a sequential mixed‐methods design, as data saturation was achieved, with no new themes emerging in the final interviews. This is consistent with methodological guidance suggesting that sample sizes between 12 and 20 participants are adequate for in‐depth thematic analysis when the aim is to achieve conceptual depth rather than generalisability [6, 7]. These participants (see Table 1) included both general nurses and mental health nurses, with varied experience levels (ranging from less than 5 years to more than 5 years) and a mix of educational backgrounds (with and without postgraduate degrees). Overall, the sample was balanced in terms of gender and professional experience within the addiction services field.

**TABLE 1:** Participants’ demographics.

Participant (pseudonym)	Gender	Education	Postgraduate degree	Position/role (deidentified)	Years of experience in addiction	Country of work
Participant 1	M	General nurse	No	Inclusion health nurse	< 5 years	Western Europe
Participant 2	M	Mental health nurse	No	Advanced practice addiction nurse	5–10 years	Portugal
Participant 3	F	General nurse	No	Senior nurse manager	> 20 years	Western Europe
Participant 4	M	Registered nurse	No	Nurse in a secure healthcare setting	> 15 years	Western Europe
Participant 5	F	General nurse	Yes	Senior nurse manager/Advanced nurse practitioner	> 20 years	Western Europe
Participant 6	M	Mental health nurse	Yes	Advanced practice addiction nurse	> 15 years	Western Europe
Participant 7	M	General nurse	No	Staff nurse in addiction services	< 1 year	Southern Europe
Participant 8	M	Mental health nurse	Yes	Senior nurse in addiction services	> 30 years	Western Europe
Participant 9	F	Mental health nurse	Yes	Nurse in a secure healthcare setting	1 year	Western Europe
Participant 10	M	Mental health nurse	Master’s	Addiction and mental health nurse	4.5 years	Western Europe
Participant 11	F	General nurse	No	Community nurse	< 5 years	Northern Europe
Participant 12	F	General nurse and Midwife	No	Staff nurse	< 5 years	Western Europe
Participant 13	M	Mental health nurse	Yes	Senior nurse manager	< 5 years	Western Europe
Participant 14	F	General nurse	No	Senior inclusion health nurse manager	> 15 years	Western Europe
Participant 15	F	General nurse	Yes	Primary care nurse manager	> 10 years	Western Europe

### 2.3. Data Collection

Data for the qualitative phase were collected through semistructured interviews conducted via Zoom between May 30th and September 18th, 2021. The interviews were designed to explore the participants’ experiences with VT, coping mechanisms and perceptions of leadership in the context of addiction services. Interview questions were developed based on the results from the initial quantitative survey, focussing on areas such as coping strategies, support systems and leadership practices. Interviews were recorded, transcribed verbatim and managed using MAXQDA Analytics Pro 2020.

### 2.4. Ethical Considerations

This study received ethical approval from the Trinity College Dublin School of Nursing and Midwifery, Faculty of Health Sciences Research Ethics Committee, on November 10th, 2020 (Ref. No. 2020902). All participants provided written informed consent prior to participating in the interviews. They were fully informed about the study’s aims, procedures and their right to withdraw from the study at any time without penalty. Confidentiality was maintained throughout the research process by pseudonymising participant data and securely storing all transcripts, recordings and other research materials. The identities of participants were kept anonymous, and their data were used exclusively for the purposes of the study.

### 2.5. Data Analysis

The qualitative data were analysed using thematic analysis based on the framework outlined by Braun and Clarke [7]. The analysis involved identifying patterns and themes within the transcribed interview data, with an emphasis on inductive coding to allow themes to emerge naturally from the data. The MAXQDA Analytics Pro 2020 [8] software was employed to assist with data management, coding and theme identification. This process provided a deeper understanding of the experiences of addiction nurses, particularly regarding the impact of leadership and organisational support in mitigating VT. The findings from the qualitative analysis were integrated with the quantitative results to inform the development of evidence‐based guidelines for organisational leadership aimed at minimising VT in addiction services.

To enhance methodological rigour, several strategies were employed throughout the qualitative phase. Reflexivity was maintained through ongoing reflection by the primary researcher regarding personal assumptions, professional background and potential influence on data interpretation during coding and theme development. The coding process was conducted systematically using an inductive approach [7], whereby transcripts were read repeatedly, initial codes were generated, and themes were refined iteratively. Coding and theme development were reviewed and discussed among the research team to enhance analytical consistency and reduce interpretive bias. Trustworthiness was further supported through the maintenance of an audit trail documenting analytical decisions, coding development and theme refinement throughout the research process. Credibility was strengthened through peer debriefing and regular supervisory discussions during data analysis.

### 2.6. Development of Organisational Guidelines

The organisational guidelines were developed using the integrated findings derived from the explanatory sequential mixed‐methods design employed in this study. Initially, the quantitative phase identified the prevalence of VT among nurses working in addiction services, together with associated risk, protective and leadership‐related factors. These findings informed the development of the semistructured interview questions used during the qualitative phase.

The qualitative phase enabled a deeper exploration of the quantitative findings and generated themes relating to nurses’ experiences of VT, coping strategies, organisational support and leadership practices within addiction services. Thematic analysis following Braun and Clarke’s [7] framework was used to identify recurring patterns and themes across the interview data.

Following completion of both phases, the quantitative and qualitative findings were integrated through the use of a joint display approach [9] (see Table 2). This process enabled direct comparison and synthesis of the findings across both datasets and facilitated the generation of meta‐inferences. The qualitative themes were subsequently translated into organisational guideline components by identifying recurring leadership and organisational factors perceived by participants as protective against VT. Themes related to supportive leadership, communication, education, supervision, staff wellbeing and organisational culture were grouped into practical evidence‐based recommendations for organisational leadership within addiction services.

**TABLE 2:** Joint display.

Quantitative finding	Qualitative finding (Illustrative quote)	Meta‐inference	Guideline component
**88.4%** of participant were at moderate/high risk to develop VT	Fourteen out of 15 participants had never heard of VT before participating in the study. *“It’s insane that we haven’t talked about it more.”* (Participant 15)	VT is highly prevalent but poorly recognised within addiction services, highlighting the need for awareness initiatives.	**Awareness of VT**
Low scores for leadership and mission (**mean = 3.3; SD = 0.877**) indicated organisations were not proactively addressing VT.	*“The organisation itself didn’t value our trauma.”* (Participant 7)	Organisations should acknowledge VT as a legitimate occupational hazard and normalise discussions around its impact.	**Normalisation of VT**
Low scores for employee empowerment and work environment (**mean = 3.1; SD = 0.792**) and staff health and wellness (**mean = 2.6; SD = 0.741**).	Nurses reported feeling undervalued and unsupported by their organisations.	Creating psychologically safe and supportive work environments is essential for reducing VT risk.	**Provide an adequate working environment**
Significant levels of VT were identified across the sample.	Participants described emotional exhaustion, intrusive thoughts, and difficulties processing trauma exposure. *“I’m extremely tired… both physically and mentally.”* (Participant 1)	Organisations should equip staff and leaders to identify and respond to early indicators of VT.	**Recognising early warning signs**
Training and professional development scores indicated inconsistent organisational support (**mean = 3.2; SD = 0.685**).	Participants repeatedly requested education on VT and coping strategies. *“It was a real eye opener.”* (Participant 3)	Education is necessary to improve understanding, prevention, and management of VT.	**Education and training**
Leadership‐related factors were associated with VT outcomes, with management and supervision showing a significant relationship with VT (**p** ** = 0.044**).	*“Like a lighthouse.”* (Participant 12); *“Communication is really important because then it makes them feel less alone.”* (Participant 9)	Visible, supportive and collaborative leadership acts as a protective factor against VT.	**Collaborative leadership approach**
Management and Supervision received the lowest organisational score (**mean = 2.5; SD = 0.704**) and was significantly associated with VT (**p** ** = 0.044**).	Participants consistently reported a lack of debriefing and regular supervision.	Regular supervision and structured debriefing are essential organisational supports for trauma‐exposed staff.	**Supervision and debriefing**
Lower organisational scores were observed across support, wellness, and management domains.	Nurses highlighted staffing shortages, limited resources and insufficient time for reflection and support activities. *“If we had the time and resources…”* (Participant 12)	Organisations should allocate sufficient staffing, time and resources to support staff wellbeing and mitigate VT.	**Allocate adequate time and resources**

*Note:* This table presents selected examples extracted from the joint display developed during the primary mixed‐methods doctoral study. Quantitative findings were integrated with qualitative themes and participant narratives to generate meta‐inferences, which informed the development of the organisational leadership guidelines. Only selected examples relevant to the organisational guidelines are presented here.

The quantitative findings also contributed to the guideline development process by highlighting areas of organisational vulnerability and leadership‐related factors associated with VT. These findings helped prioritise the organisational domains addressed within the guidelines and supported the relevance of the qualitative themes identified.

The development of the guidelines followed an evidence synthesis approach based on the integration of quantitative findings, qualitative themes, and relevant literature identified within the study. The process was further informed by the Health Service Executive (HSE) Knowledge Translation Framework [10], which supports the translation of research evidence into actionable recommendations for practice. Following the generation of meta‐inferences, the integrated findings were synthesised into practical organisational leadership recommendations addressing awareness, education, leadership practices, supervision, support and staff wellbeing within addiction services. No formal external validation process was conducted; however, the guidelines were developed directly from the integrated findings of both research phases to ensure alignment with participants’ experiences and the empirical evidence generated by the study.

Although guidelines were developed at both organisational and individual levels, this paper focuses exclusively on the organisational leadership guidelines.

## 3. Results

Thematic analysis of the qualitative interviews revealed five overarching themes and thirteen subthemes (see Table 3), which together capture the nuanced experiences of nurses working in addiction services and their encounters with VT. The findings highlight both the challenges and opportunities for growth inherent in this demanding field, as well as the systemic and interpersonal factors that mediate nurses’ experiences. These themes encompassed (1) positive growth associated with VT, (2) recognising negative consequences of VT, (3) the role of leadership, (4) What kind of support is required?, and (5) the rise of the “addiction nurse.”

**TABLE 3:** Themes and subthemes.

Themes	Theme 1 Positive growth associated with VT	Theme 2 recognising negative consequences of VT	Theme 3 the role of leadership	Theme 4 what kind of support is required?	Theme 5 the rise of “the addiction nurse”
	“Where do I store this trauma?”	“Sink or swim”	“Like a lighthouse”	“I need your help.”	“We try not to fail”
Subthemes	• Awakening awareness of VT • Changes and personal positive growth • Qualities needed for working in the addiction field.	• Adverse consequences in working with traumatised patients. • Through personal qualities	• Nurses’ participation in leadership • Collective work • Importance of leadership in VT ‐ Positive communication and VT	• Social, organisational and peer support • Poor health organisations	• Choosing this specialisation • Significance of the job • Challenges

### 3.1. Theme 1: Positive Growth Associated With VT—“Where Do I Store This Trauma?”

The first theme captures the paradoxical experience of nurses who, despite frequent and intense exposure to patient trauma, reported unexpected psychological and professional growth. Three subthemes were identified as follows: awakening awareness of VT, changes and personal positive growth, and qualities needed for working in the addiction field.

A striking finding was that 14 of the 15 participants had never encountered the concept of VT prior to participating in this study. This lack of prior awareness aligns with existing research indicating that VT is often poorly understood or underacknowledged among healthcare professionals, leading to missed opportunities for prevention and early intervention [11, 12]. For participants, the interview process itself acted as an educational experience. Participant 3 described the study as *“a real eye opener,”* while Participant 15 expressed surprise and concern that *“it’s insane that we haven’t talked about it more.”* In a few cases, this new awareness prompted participants to raise the issue within their own organisations, resulting in tangible structural changes such as increased supervision availability and dedicated team discussions on the emotional impact of clinical work. This suggests that increased awareness of VT can translate into early organisational reflection and small practice changes.

Yet awareness also brought emotional complexity. Participant 15’s rhetorical question—*“Where do I store this trauma?”*—conveyed the difficulty of carrying patients’ distress while lacking clear pathways for processing it. This illustrates the initial shock and emotional toll that can accompany recognition of VT. Over time, participants described beginning to develop coping strategies, suggesting a process of adaptation and resilience‐building. Participant 1, for example, explained how he learned to regulate his emotional responses by focussing on controllable factors: *“That helped me… to deal with patients’ trauma.”* This reflects developing emotional regulation skills as part of professional adaptation [13].

Other participants, like Participant 12, acknowledged becoming “*a little bit desensitised*,” a defence mechanism often identified in the literature as emotional numbing [14, 15]. Although often viewed negatively, participants described this as a protective strategy that helped them cope with repeated exposure to trauma.

Participants also identified specific qualities they believed were essential for sustained work in addiction services: empathy, compassion, patience and firm boundary‐setting. Santiago’s statement—*“I am your friend here… but if you try to manipulate me, I will tell you ‘Stop’”*—captures the delicate balance between emotional connection and professional detachment. These findings echo earlier research by Wilson and Lindy [16] and Rydon [17], which emphasise the dual role of empathy as both a therapeutic strength and a potential source of vulnerability in clinical care.

Collectively, this theme suggests that while VT awareness may initially provoke discomfort or self‐doubt, it can also catalyse personal growth, strengthen professional identity, and promote the development of adaptive coping skills. However, this growth appears to depend on both individual reflection and supportive organisational environments.

### 3.2. Theme 2: Recognising Negative Consequences of VT—“Sink or Swim”

This theme captured participants’ awareness of the personal and occupational risks inherent in working with traumatised individuals, highlighting both the adverse consequences of client trauma exposure and the influence of personal factors on their professional wellbeing. Participants often described experiences consistent with symptoms widely recognised in the VT literature—such as burnout, intrusive thoughts, emotional exhaustion and a gradual erosion of emotional reserves [1, 18]. These experiences were often described as overwhelming and cumulative in nature. Participant 9 encapsulated the stark reality of the work with a succinct metaphor: “You either sink or swim.” This reflects a perception that nurses must rely on individual coping rather than structured organisational support. For her, the phrase reflected the absence of middle ground‐workers either found ways to cope and adapt or risked being overwhelmed by the cumulative demands of their role. Similarly, Participant 1 offered a candid account of the toll this work had taken: “I’m extremely tired… both physically and mentally.” This highlights how VT affects both emotional and physical wellbeing, reinforcing its chronic and draining nature. His words echoed a recurrent sentiment that the continuous exposure to clients’ trauma stories was not only mentally draining but also physically depleting.

Personal histories of trauma also emerged as a powerful influence on participants’ experiences. For some, these histories appeared to serve as a source of resilience. Participant 10 reflected on his own childhood loss, describing how it fostered a sense of inner strength: “It built in a strength into myself… it helps me.” This suggests that previous trauma experiences may enhance coping capacity and emotional understanding in clinical practice. His perspective resonates with evidence suggesting that personal trauma can sometimes support empathy and resilience [19].

In contrast, other participants described how personal histories made them more susceptible to distress. Participant 5 recounted experiencing intrusive dreams that mirrored her work, particularly when client narratives overlapped with her own life circumstances: “I was always dreaming that social care would take away my baby.” Such accounts highlight what McCann and Pearlman (1990) describe as the “permeability of professional boundaries” in VT—where the separation between personal and professional worlds becomes blurred.

Collectively, these experiences underscore that VT is not experienced in a uniform way but it is shaped by the interaction between occupational exposure and personal history. They also highlight the urgent need for organisational safeguards, including regular supervision, opportunities for reflective practice, and access to psychological support. Without these protective structures, the risk of “sinking” rather than “swimming” remains a significant and ongoing concern for those working in trauma‐exposed roles.

### 3.3. Theme 3: The Role of Leadership—“Like a Lighthouse”

Leadership emerged as a pivotal factor in shaping how VT was experienced and managed, operating as both a protective buffer and a potential stressor in relation to its effects. Three interconnected subthemes were identified: *nurses’ participation in leadership; collective work, and Importance of leadership in VT; positive communication and VT.* Across accounts, effective leadership was not confined to a traditional top–down model; rather, it was often described as relational, situational and shared among team members. This reflects a distributed leadership approach, where responsibility and influence are shared across the team [20, 21].

Participant 12 captured the protective role of leadership through a vivid metaphor: *“like a lighthouse,”* she said, describing leaders as beacons of steadiness who offer guidance, visibility and reassurance during times of uncertainty. This suggests that visible and consistent leadership provides emotional safety for staff working in high‐stress environments. Such leaders provided clarity of direction while ensuring that staff felt supported in navigating the emotional demands of trauma work.

For some, effective leadership was not a title but an attitude; it was enacted in everyday practice. Participant 5 described leadership as *“walking beside the patient,”* positioning it as a compassionate and empowering act grounded in empathy and presence rather than authority. This highlights leadership as relational and supportive rather than hierarchical. Participant 15 and Participant 13 echoed this ethos, with Participant 13 emphasising authenticity and self‐reflection: *“If I’m expecting my staff to explore trauma… I need to do that myself.”* This notion of leading by example was seen as vital for fostering trust and psychological safety within teams [22, 23].

Participants consistently identified communication, trust and accessibility as hallmarks of strong leadership. Leaders who listened, responded openly to concerns and were visibly engaged in the realities of frontline work were perceived as creating a safer emotional climate. This suggests that relational communication is a key protective factor against VT. In contrast, poor leadership—characterised by distance, lack of transparency or limited empathy—was associated with increased emotional strain, weakened morale and fractured team relationships, all of which heightened vulnerability to VT [24].

These accounts suggest that leadership in trauma‐exposed work settings extends beyond operational management; it is about modelling resilience, facilitating collective coping, and ensuring that no team member feels adrift in the demanding waters of trauma care. In this sense, effective leaders, much like Participant 12’s lighthouse, provide not only direction but also a psychological anchor, helping teams weather the storms inherent in their work.

### 3.4. Theme 4: What Kind of Support is Required?—“I Need Your Help”

This theme explored the range and quality of support systems available to nurses working in trauma‐exposed settings, focussing on two subthemes: social, organisational and peer support, and health organisation support. Overall, participants highlighted peer support as the most immediate and accessible form of emotional assistance.

Participants’ accounts consistently highlighted the vital role of peer support as an immediate and accessible buffer against the emotional demands of the work. For many, colleagues were the first point of contact in moments of distress, offering both practical guidance and emotional validation. Participant 5 illustrated this reliance vividly: *“I knock on their door and say, ‘Do you have time for me because I need your help.’”* This reflects the importance of informal, real‐time peer support in managing emotional distress in clinical settings. Her words conveyed not only the urgency of the need but also the trust embedded in these professional relationships.

Similarly, Participant 15, Participant 4 and Participant 12 emphasised that peer connections were more than a source of casual camaraderie; they functioned as a key source of emotional stability. This suggests that peer relationships play a protective role in reducing the emotional impact of VT. This sentiment is strongly supported in the literature, which identifies collegiality as a protective factor in trauma‐exposed healthcare environments [25].

However, when participants turned their focus to organisational support, a contrasting narrative emerged. Many reported systemic shortcomings, describing formal structures as either inadequate or entirely absent. Santiago’s experience captured this frustration: *“The organisation itself didn’t value our trauma.”* His statement reflects a perceived disconnect between the frontline reality of trauma care and the institutional recognition of its emotional toll.

Participant 4 recalled a particularly stark example of insufficient support, describing how after a distressing incident, he received no formal debriefing—only the dismissive instruction: *“They just said, ‘Yeah, grand, get a cup of tea.’”* This illustrates the absence of structured debriefing processes and reliance on informal coping responses. Such experiences reinforce concerns documented in previous research, which points to a lack of structural safeguards and a tendency for healthcare systems to rely on informal coping mechanisms rather than embedded, proactive measures [26].

While a small number of nurses had access to external supervision, employee assistance programmes or ad hoc mental health supports, these were often self‐initiated, inconsistently applied or not tailored to the unique demands of addiction care. The absence of formalised processes left staff to navigate the psychological aftermath of traumatic encounters largely on their own. Participants voiced a strong and recurring call for improvements, advocating for mandatory and routine supervision, structured debriefing after critical incidents and enhanced education on the impacts of trauma exposure.

These recommendations align with existing evidence emphasising the importance of proactive organisational interventions in protecting staff wellbeing [27]. Overall, participants clearly identified a gap between informal peer support (strong) and formal organisational support (weak), highlighting the need for more structured systems of care within addiction services.

### 3.5. Theme 5: The Rise of the Addiction Nurse—“We try not to Fail”

The final theme explored the evolving professional identity of nurses in addiction services, capturing both the personal journeys that led participants into the field and the challenges and rewards that shape their ongoing commitment. Three subthemes were identified: *choosing this specialisation, significance of the job* and *challenges.*

For most, entry into addiction nursing was neither deliberate nor anticipated. As Participant 14 put it bluntly, *“It wasn’t something I wanted to do… definitely not.”* This reflects initial reluctance or lack of intention to enter addiction nursing as a speciality. This sentiment reflected a broader pattern in which participants described arriving in the field through chance placements, staffing needs or career transitions rather than a planned vocational choice.

However, over time, initial ambivalence gave way to a profound sense of purpose. Tiago captured this dedication with the succinct declaration: *“We try not to fail,”* framing the work as a commitment not only to professional standards but also to the dignity and humanity of their patients. Participant 1’s reflection—*“If it wasn’t for me, no one would be looking at them”*—highlighted the advocacy role many nurses embraced, positioning themselves as essential voices for a patient group often marginalised within the wider healthcare system. These experiences resonate with Rushton et al. [28] findings, which identify intrinsic motivation, moral commitment, resilience and a strong sense of professional responsibility as factors sustaining nurses in high‐intensity clinical settings.

The role, however, is far from without frustration. Participants described a healthcare landscape constrained by limited resources, insufficient multidisciplinary collaboration and high relapse rates that could erode morale. Participant 1 lamented the persistent *“lack of options”* available to clients, pointing to service gaps in rehabilitation, housing and aftercare. Participant 12 drew a direct connection between staffing shortages and the prevalence of challenging patient behaviours: *“If we had the time and resources… the challenging behaviours would be reduced.”* These insights underscore the systemic pressures that not only affect patient outcomes but also contribute to nurse burnout and compassion fatigue.

Despite these obstacles, a strong sense of purpose and professional pride prevailed. Participants repeatedly described small patient successes—whether a client attending treatment consistently, securing stable housing or reconnecting with family—as deeply rewarding milestones. Such moments were framed not as isolated victories, but as affirmations of the nurse’s role in sustaining hope and fostering recovery.

In recognising these contributions, the data pointed to an urgent need for organisational acknowledgement of addiction nursing as a specialised and skilled discipline within healthcare. Supporting these nurses through targeted training, adequate staffing and integrated care pathways would not only improve patient outcomes but also reinforce the resilience and retention of those who, as Tiago put it, “try not to fail.”

## 4. Discussion

This qualitative phase of the study provides an in‐depth exploration of VT among nurses working in addiction services, revealing the complex interplay between personal resilience, professional growth and systemic support in trauma‐exposed healthcare environments. Participants’ limited prior awareness of VT, reported by 14 of 15 nurses, underscores persistent gaps in professional training and organisational culture, echoing earlier findings that highlight the pervasive under recognition of VT in clinical settings [11, 12]. Participation in this research appeared to catalyse reflection, dialogue and organisational responsiveness, supporting Bride’s [1] assertion that recognition alone can be an initial step toward trauma‐informed practice.

These qualitative findings expanded upon the quantitative phase, which identified low levels of awareness and organisational preparedness regarding VT among nurses working in addiction services. The interviews provided deeper insight into how this lack of awareness affected nurses’ ability to recognise, process and respond to VT within their professional roles.

The findings suggest that VT among addiction nurses operates as a multidimensional phenomenon shaped by repeated trauma exposure, emotional labour and organisational context. While participants described significant psychological strain, they also demonstrated adaptive coping and professional growth, reinforcing the complex relationship between vulnerability and resilience within trauma‐exposed nursing environments. At the same time, participants reported adaptive growth, including improved emotional regulation, better boundary‐setting and strengthened empathy, consistent with Tedeschi and Calhoun’s [13] theory of posttraumatic growth and evidence of vicarious posttraumatic growth [19, 29]. These findings underscore the complex tension between vulnerability and resilience, suggesting that personal coping strategies, while valuable, are insufficient without systemic reinforcement. Leadership emerged as a central organisational determinant influencing how nurses experienced and managed VT. The findings extend existing trauma‐informed leadership literature by demonstrating that visible, relational and psychologically supportive leadership practices may strengthen resilience and reduce emotional burden within addiction services [2, 30].

This finding directly supports the quantitative results, where leadership‐related factors were significantly associated with VT risk. The qualitative narratives further explained how supportive, visible and communicative leadership practices were perceived as protective against the emotional impact of trauma exposure.

Participants emphasised the importance of trust, communication, accessibility and role‐modelling, demonstrating that leadership can influence both team cohesion and the emotional climate, which in turn affects vulnerability to VT. Yet, despite the potential protective role of leadership, systemic supports were often lacking. Nurses reported insufficient organisational debriefing, minimal access to formal supervision and a reliance on informal peer support, reflecting broader concerns regarding structural neglect in healthcare institutions [26, 27, 31].

The findings also emphasise the importance of relational support structures within trauma‐intensive workplaces. Informal peer support appeared to compensate for deficiencies in formal organisational systems, suggesting that healthcare organisations may rely excessively on collegial coping mechanisms in the absence of structured trauma‐informed interventions [25]. The study additionally highlights the evolving professional identity of addiction nurses, characterised by strong moral commitment, advocacy and emotional investment despite ongoing systemic pressures. This reinforces the need for greater organisational recognition of addiction nursing as a specialised trauma‐exposed field requiring targeted leadership and workforce supports.

While the quantitative findings highlighted support systems as an important protective factor, the qualitative phase expanded this understanding by illustrating how informal peer relationships often compensated for the absence of formal organisational supports.

Simultaneously, systemic frustrations, such as under‐resourcing, high relapse rates and inadequate discharge planning, were reported to exacerbate stress and hinder effective care, suggesting that individual resilience alone cannot compensate for structural deficiencies. Taken together, these findings illustrate that VT in addiction nursing is a multifaceted phenomenon, shaped by individual, relational and organisational factors. They reinforce the need for multilevel interventions, including comprehensive VT education, routine supervision and debriefing, active and distributed leadership, peer support integration and recognition of the specialised contributions of addiction nurses. Such interventions are critical not only to safeguard nurses’ wellbeing but also to sustain the quality of care provided to traumatised populations.

### 4.1. Conceptual Contribution and Novelty

This study makes a conceptual contribution to the growing literature on trauma‐informed leadership by situating VT within addiction nursing practice using a sample of European nurses, thereby extending evidence beyond the predominantly North American and general mental health contexts in which VT has traditionally been examined [32–34]. In doing so, the findings reposition VT not as an individual or purely psychological issue located within the nurse but as a workplace‐ and system‐related phenomenon embedded within organisational culture, leadership practices and service demands. This represents an important conceptual shift from earlier research that has largely emphasised individual coping, burnout and resilience, toward a more occupational and organisational framing of VT [35, 36].

In contrast to much of the existing literature that foregrounds the predominantly negative consequences of VT, the findings from this study also highlight the presence of adaptive and protective coping processes, including peer support, reflective practice and meaning‐making within clinical work. This more nuanced understanding demonstrates that VT is not solely a deficit‐based construct but can also be associated with professional growth when supported appropriately within the workplace context. Importantly, the study further identifies organisational leadership as a central determinant in shaping these experiences, acting both as a protective factor and, in some contexts, a source of vulnerability depending on visibility, support structures and communication practices.

The findings also demonstrate that addiction nurses face distinctive emotional and relational demands associated with repeated exposure to complex trauma, relapse, stigma and long‐term patient suffering, reinforcing the need for leadership approaches that are proactive, relational, visible and explicitly trauma‐informed. The use of a knowledge translation approach strengthened the applied value of the study by ensuring that the integrated mixed‐methods findings were systematically translated into actionable organisational leadership recommendations.

Furthermore, the empirically informed organisational leadership guidelines developed in this study differ from existing frameworks by being directly grounded in the integrated quantitative and qualitative experiences of European addiction nurses, rather than being adapted from broader trauma‐informed or mental health models. As such, the study offers a context‐specific, empirically grounded contribution to nursing leadership knowledge and provides a practical foundation for developing sustainable trauma‐informed organisational cultures within addiction services.

## 5. Implications for Nursing Management

The findings from this study highlight the critical imperative for nursing management and organisational leadership to adopt a structured, evidence‐informed and strategically integrated approach to mitigating VT among nurses, particularly in addiction services and other trauma‐intensive care settings. Table 4 synthesises a set of organisational guidelines derived directly from international literature, and this study insights, translating empirical evidence into actionable, leadership‐focused strategies.

**TABLE 4:** Organisational guidelines.

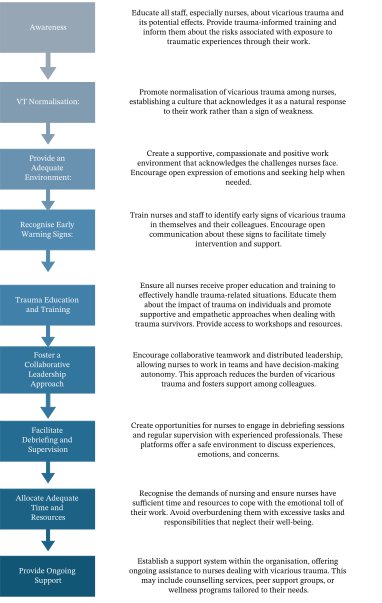

To enhance clarity and feasibility of implementation, four key leadership actions are prioritised.

First, leadership‐led awareness and normalisation of VT as an occupational response should be embedded within organisational communication and staff development strategies. This involves explicitly acknowledging VT in policy documents, routine team discussions and induction programmes to reduce stigma and encourage early recognition of stress responses among staff.

Second, structured reflective practice and clinical supervision systems should be implemented and protected at organisational level. This includes scheduled, formalised debriefing sessions following exposure to distressing clinical events and ensuring access to trained trauma‐informed supervisors who can support emotional processing and professional reflection.

Third, organisations should develop and sustain peer support mechanisms and psychologically safe team cultures. This can be operationalised through peer support champions, facilitated reflective groups and protected time for informal team check‐ins that promote shared coping and collective resilience.

Fourth, leadership development should incorporate trauma‐informed and distributed leadership competencies. This requires targeted training for nurse managers focussing on recognising VT, responding supportively to staff distress and fostering inclusive decision‐making environments that prioritise staff wellbeing alongside service delivery.

The implementation of these strategies carries significant implications for both staff wellbeing and organisational outcomes. VT has been consistently associated with negative psychological consequences for nurses, including heightened stress, emotional exhaustion and diminished professional efficacy [1, 26, 27]. Left unaddressed, these consequences can exacerbate turnover, compromise patient care quality and erode team cohesion. Conversely, evidence‐based interventions—such as distributed leadership, structured debriefing protocols, access to trauma‐informed supervision and peer support frameworks—have demonstrated measurable reductions in emotional burden, enhanced collaborative functioning and improvements in job satisfaction [2, 20, 21].

Critically, fostering an organisational culture that openly acknowledges and normalises VT serves as both a preventative and restorative strategy. Normalisation reduces stigma, facilitates early identification of stress responses, and encourages proactive help‐seeking behaviours, thereby mitigating the progression toward burnout or compassion fatigue. Nursing managers who operationalise these principles function as “emotional stewards,” balancing operational imperatives with the psychosocial needs of staff while promoting psychologically safe environments.

From a strategic standpoint, integrating these evidence‐informed approaches represents a proactive investment in organisational resilience and workforce sustainability. By embedding VT mitigation into leadership practice, organisations not only safeguard nurses’ mental health but also reinforce the delivery of high‐quality, patient‐centred care within complex, high‐risk clinical environments. Failure to address VT systematically risks both human and organisational capital, whereas deliberate, structured leadership interventions position nursing teams to maintain performance, resilience and professional satisfaction over the long term.

## 6. Conclusion and Recommendations

This paper highlighted the lived experiences of European nurses working in addiction services to understand the impact of VT and the role of organisational leadership in mitigating its effects. Five key themes emerged from the data: positive growth associated with VT, the recognition of its negative consequences, the central role of leadership, the types of support required and the evolving professional identity of addiction nurses. These findings illustrate the dual nature of VT—as both a risk and an opportunity for growth—and underscore how leadership can function as either a protective buffer or an exacerbating factor, depending on its approach and visibility.

From a nursing management perspective, the study highlights the urgent need to acknowledge VT as a significant occupational hazard in addiction services. Effective, proactive leadership is not a luxury but a necessity in trauma‐laden environments. Nurse managers must take an active role in fostering emotionally supportive work cultures, implementing trauma‐informed practices and ensuring that staff are not left to manage the emotional toll of their work in isolation. Leadership should be seen as a shared, relational responsibility that requires empathy, presence and responsiveness.

To support this shift, the study developed the first preliminary evidence‐based organisational leadership guidelines specifically aimed at addressing VT in addiction nursing. These guidelines are grounded in the qualitative narratives of addiction nurses, the quantitative findings from the first phase of the study and existing theoretical frameworks. Key elements include (1) awareness; (2) normalisation; (3) education; (4) adequate resources and (5) ongoing support. These guidelines offer a practical roadmap for healthcare organisations to move from ad hoc responses to sustainable, systemic support for nurses at risk of VT.

Future research should focus on piloting and evaluating the effectiveness of these guidelines in real‐world settings. Longitudinal studies are needed to assess whether structured leadership interventions reduce VT symptoms, improve staff wellbeing and enhance patient care outcomes over time. Additionally, further studies should explore the perspectives of nurse managers, multidisciplinary team members and service users to build a comprehensive model of organisational resilience in addiction services.

In conclusion, this study fills a critical gap in the literature by centring the voices of addiction nurses and translating their insights into actionable leadership strategies. By prioritising emotional safety and trauma‐informed leadership, nursing management can not only protect their staff but also strengthen the quality and sustainability of addiction care services.

## 7. Study Limitations

Some limitations should be acknowledged. The qualitative sample was small and purposively selected from a self‐selected group, introducing potential self‐selection bias as participants may have been more engaged or interested in the topic of VT. While the findings offer rich insights, they may not be generalisable to all addiction nurses across Europe due to cross‐country variability in healthcare systems, organisational structures and service provision, which may influence experiences of VT and leadership support. Moreover, interviews were conducted during the COVID‐19 pandemic, which may have heightened experiences of stress, trauma and leadership gaps, influencing participants’ perspectives. However, mitigating this limitation was the depth and breadth of experience of the participants within a range of addiction services.

The study also focused solely on nursing perspectives and did not capture views from multidisciplinary team members. Including these voices could provide a more holistic understanding of organisational dynamics. Finally, while the study informs leadership guideline development, future research should evaluate the real‐world implementation and effectiveness of these guidelines in reducing VT.

## Author Contributions

**Kristine Nicki Annunziata:** conceptualisation, methodology, investigation, data curation, formal analysis, writing–original draft and visualisation. **Elizabeth Curtis:** supervision, methodology, validation and writing–review and editing. **Catherine Comiskey:** supervision, methodology, formal analysis, validation and writing–review and editing.

## Funding

No external funding was received from any public, commercial or not‐for‐profit funding agency.

## Disclosure

This research was conducted as part of a self‐funded Doctor of Philosophy programme.

## Ethics Statement

Ethical approval was obtained from the Trinity College Dublin School of Nursing and Midwifery, Faculty of Health Sciences Research Ethics Committee (Reference No. 2020902), which was approved on 10 November 2020.

## Conflicts of Interest

The authors declare no conflicts of interest.

## Data Availability

The datasets generated and analysed during the current study are not publicly available due to the confidential nature of the participant data but are available from the corresponding author on reasonable request.
